# Predictors of Oral Health in Young Swedish Adults: A Register-Based Observational Study

**DOI:** 10.1155/ijod/9914605

**Published:** 2025-10-28

**Authors:** Jessica Skoogh Andersson, Anna Trullenque-Eriksson, Peter Lingström, Jan Derks

**Affiliations:** ^1^Department of Periodontology, Institute of Odontology, The Sahlgrenska Academy, University of Gothenburg, Gothenburg, Sweden; ^2^Department of Cariology, Institute of Odontology, The Sahlgrenska Academy, University of Gothenburg, Gothenburg, Sweden

## Abstract

**Aim:**

This retrospective registry study aimed to identify psychosocial and behavioral predictors of oral health in young adulthood.

**Materials and Methods:**

A total of 335 Swedish individuals who completed questionnaires pertaining to oral health-related attitudes, perceptions, and behaviors at 19 years, were followed through young adulthood (23–31 years) using registry data. Information on missing, decayed/filled teeth, periodontal status, and self-assessed (SA) oral health scores was retrieved. Predictors of professionally-assessed (PA) and SA oral health were explored using regression analyses.

**Results:**

Considering oral health to be very important at 19 years was a predictor for good PA oral health in young adulthood (odds ratio [OR] 3.2, 95% CI 1.1, 9.4). Females (risk ratio [RR] 4.1, 95% CI 1.8, 9.4) and those reporting taking very good care of their teeth (RR 2.5, 95% CI 1.2, 5.3) were more likely to later report very good SA oral health. Smoking increased the risk for poor–very poor SA oral health (RR 5.1, 95% CI 1.9, 13.8). Few participants brushed infrequently at 19 years and they were polarized in their later SA oral health scores.

**Conclusions:**

A positive attitude towards oral health at age 19 years was associated with good PA oral health in young adulthood, whereas gender, smoking, toothbrushing frequency, and the perceived quality of own dental care were predictors of subsequent SA oral health.

## 1. Introduction

Oral diseases are highly prevalent, dominated by dental caries and periodontitis. It is estimated that approximately 2 billion individuals globally suffer from untreated caries in the permanent dentition [1, 2]. The corresponding estimate for severe periodontitis is approximately 1 billion [3, 4].

Both caries and periodontitis are multifactorial diseases mainly caused by bacteria. The interaction over time between cariogenic bacteria, fermentable carbohydrates, and tooth substance determines whether a local destruction of dental tissues will occur. In the case of periodontitis, it is the interplay between the microbial plaque and the host response that determines if and to what degree soft tissue inflammation will progress into breakdown of tooth-supporting structures. A variety of risk factors, either biological/genetic, behavioral or lifestyle-related, have been identified for both diseases [5–8].

Beyond specific oral diseases, oral health is increasingly recognized as a broader concept. For instance, according to the FDI World Dental Federation, oral health is multifaceted and should include the ability to speak, smile, smell, taste, touch, chew, swallow, and convey a range of emotions through facial expressions with confidence and without pain, discomfort, and disease of the craniofacial complex [9]. Health-related attitudes and socioeconomic parameters have been reported as risk factors related to oral health in adolescence [10–14]. Attitude has been defined as the “psychological tendency to evaluate a particular entity with some degree of favor or disfavor” [15]. Attitudes are complex and composed of cognitive (e.g., knowledge and beliefs), affective, and behavioral processes and responses [15, 16]. They are developed in social contexts, where family and friends are particularly relevant [17]. However, there is limited evidence on how risk factors and attitudes earlier in life may predict oral health in adulthood. Conventional longitudinal cohort studies are challenging, both in identifying and retaining meaningful study populations over time. Register-based studies may serve as a valuable complement in following cohorts longitudinally.

The aim of this register-based study was to identify psychosocial and behavioral predictors of oral health in young adulthood.

## 2. Materials and Methods

A previously identified cohort [11] was followed longitudinally in the present retrospective register-based study. The protocol was approved by the Swedish Ethical Review Authority (ref no: 2019-02306) and STROBE guidelines were followed in the reporting.

### 2.1. Patient Sample and Data Extraction

Initially, a total of 506 Swedish subjects born in 1987 were clinically examined at 19 years of age as part of a cross-sectional study [11]. Demographic and socioeconomic data (e.g., gender, parent education, and education/occupation at age 19) were retrieved and participants answered questions regarding their oral health-related behavior, perceptions, and attitudes [11]. They also completed validated questionnaires related to dental anxiety [18] and self-efficacy [19]. Anxiety was scored on a scale ranging from 5 to 20. Self-efficacy was scored from 10 to 40 with higher values representing greater self-efficacy. For details, see Ericsson et al. [11, 20]. The patient sample is described in Table 1.

For the period 2010–2018, data on oral status were retrieved from the Swedish Quality Registry for Caries and Periodontal diseases (SKaPa) and were available for 345 individuals. Information was obtained on an annual basis, including:

• Missing teeth.
• Number of teeth with manifest caries or restorations (DFT).
• Number of teeth with periodontal probing depth (PPD) ≤3 mm, PPD 4–5 mm, and PPD ≥6 mm.
• Response to the question “How do you judge your current oral health?” (very good/good/poor/very poor; included in the examination protocol within public dental health care).

Data on periodontitis (derived from PPD) and its onset have been reported elsewhere [21]. Third molars were not considered in any of the assessments.

Demographic and socioeconomic data at age 30–31 years were available for 344 of 345 individuals (Swedish Total Population Register and the Longitudinal Integrated Database for Health Insurance and Labour Market Studies, Statistics Sweden).

### 2.2. Outcomes and Statistical Analysis

We defined a composite outcome, professionally-assessed (PA) oral health (primary outcome: good PA oral health), as a DFT score of zero as well as the absence of any PPD >3 mm and no teeth lost over the entire follow-up period. Considering DFT as a continuous variable, we scored its maximum value over the follow-up to represent each individual. Similarly, periodontal health was categorized according to the most severe PPD over the follow-up (maximum PPD ≤3 mm; PPD 4–5 mm and/or 1 tooth with PPD ≥6 mm; ≥2 teeth with PPD ≥6 mm).

In addition to PA oral health, we also scored self-assessed (SA) oral health based on the self-rated question “How do you judge your current oral health?”. The participant was represented by the mode over the follow-up period; when multiple modes were available, the maximum value (worst) was chosen.

Potential predictors of PA oral health and SA oral health were evaluated through regression analyses. For the dichotomous outcome PA oral health, logistic regression was used. The model was built in a stepwise manner. Potential predictors with *p*-values <0.10 in the simple models were initially considered. When independent variables were conceptually correlated, only one of them was selected based on coefficients, *p*-values, and Akaike's information criterion. Nested models were compared through likelihood-ratio tests. Only statistically significant variables (*p*  < 0.05) were included in the final model. Linear regression analyses were used for DFT and multinomial logistic regression analyses were used for periodontal health and SA oral health. Models were built in the same manner as for PA oral health. The results of Hosmer–Lemeshow goodness-of-fit test are provided for final logistic and multinomial logistic regression models, and adjusted *R*-squared for the final linear regression model. Ten of 345 individuals with missing data on predictors were excluded. All models were adjusted for years of follow-up.

All analyses were performed in Stata (Stata SE version 18.0, StataCorp LLC, TX, USA). Categorical variables were presented as frequencies and percentages, and continuous variables as means ± standard deviation unless specified otherwise. Outcomes were reported as adjusted odds ratios (ORs; from logistic regression models), risk ratios (RRs; from multinomial regression models) and coefficients (from linear regression models) with 95% confidence intervals (95% CIs). Statistical significance was set at 0.05.

## 3. Results

In total, 335 individuals were included in the study. The follow-up period, represented by entries in the SKaPa registry, covered an average of 7.2 ± 2.7 years (range: 1–9). Demographic and socioeconomic data are provided in Table 1 (characteristics by PA and SA oral health are presented in Tables A1 and A2).

### 3.1. Age 19 Years

At 19 years, 33% reported exercising less than once per week, 11% were regular smokers (14 individuals smoked ≥10 cigarets per day) and 13% were snuff users. Seventy-five percent of the participants believed oral health to be very important and almost all considered cleaning their teeth to be important (98%). Eighty-five percent of respondents believed they took good care of their teeth (“well” or “quite well”). Additionally, 82% rated daily toothbrushing with fluoride toothpaste to be important and 79% reported brushing their teeth twice daily. While 40% considered interdental cleaning to be important, only 6% reported daily interdental cleaning using floss and/or toothpick.

Self-efficacy scores were generally high, with a majority of participants (96%) rating the possibility to influence their oral health to be large or quite large. Severe dental anxiety was not common (8%).

### 3.2. Age 30–31 Years

At 30–31 years of age, most individuals were employed (87%); 31% were married and 59% had children. The median disposable income was 2.6 times higher than the Swedish living standard (based on the family income after tax in relation to an official reasonable standard of living).

### 3.3. PA Oral Health, DFT, and Periodontal Health in Young Adulthood

Of the 335 individuals, 38 (11%) presented with good PA oral health during the entire follow-up. None lost teeth over the observation period. Twenty-one percent were free from caries (DFT range 0–19; mean 3.8 ± 3.7) and 53% did not present with PPD >3 mm at any time (10% with PPD ≥6 mm at ≥2 teeth at least once).

Individuals who did not consider their oral health to be very important at age 19 years were less likely to present with good PA oral health in young adulthood (OR 0.3, 95% CI 0.11, 0.90). No other parameters were associated with PA oral health (Table A3).

**Figure 1:**
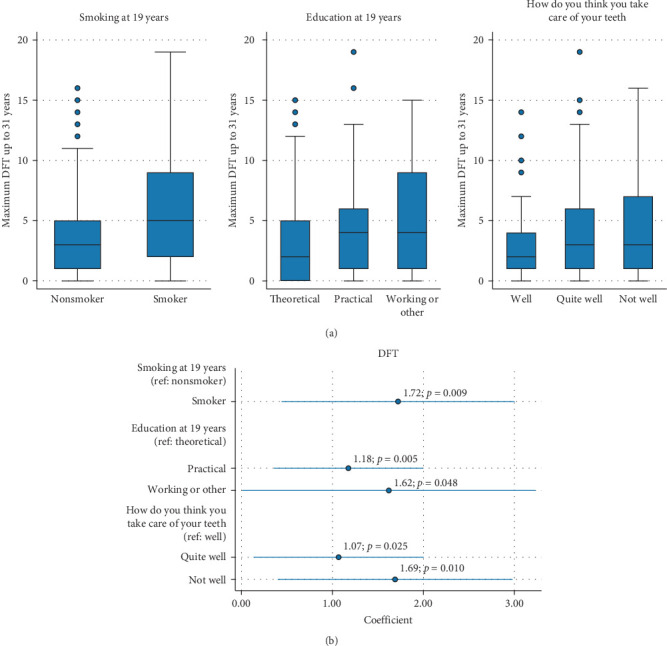
Boxplots illustrating DFT scores by smoking, education, and “How do you think you take care of your teeth” (a) and coefficients with 95% CI from the linear regression model (outcome: DFT) (b).

Smoking and education/occupation at 19 years were predictors of DFT in young adulthood, as was perception of own oral hygiene (Figure 1 and Table A4).

**Figure 2:**
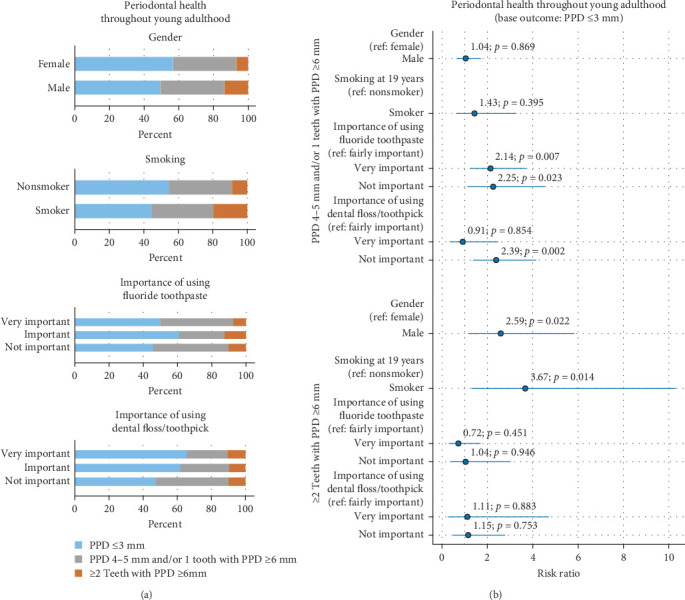
Distribution of periodontal health by gender, smoking, “Importance of using fluoride toothpaste,” and “Importance of using dental floss/toothpick” (a), and OR with 95% CI from the multinomial logistic regression model (outcome: periodontal health) (b).

Gender, smoking, and perceived importance of toothbrushing and interproximal cleaning were predictors of periodontal health. Males and smokers were more likely to present with ≥2 teeth with PPD ≥6 mm. Individuals who considered toothbrushing to be very important or not important at all and those considering interproximal cleaning not to be important were more likely to present with PPD 4–5 mm and/or 1 teeth with PPD ≥6 mm (Figure 2 and Table A5).

### 3.4. SA Oral Health in Young Adulthood

Of the 335 individuals, 227 provided data on SA oral health at least once during the 10-year period (average number of assessments 3.9 ± 1.7; range: 1–8). Oral health was self-rated as very good for 15.5%, good for 71.5%, and poor–very poor for 13% of participants.

Good PA oral health was more frequent among those with very good SA oral health (very good: 30%; good: 7%; poor–very poor: 14%). Mean DFT was higher among those assessing their oral health as poor (very good: 2.5 ± 3.4; good: 3.6 ± 3.4; poor–very poor: 5.1 ± 4.8), as was the prevalence of ≥2 teeth with PPD ≥6 mm (very good: 2%; good: 9%; poor–very poor: 17%).

**Figure 3:**
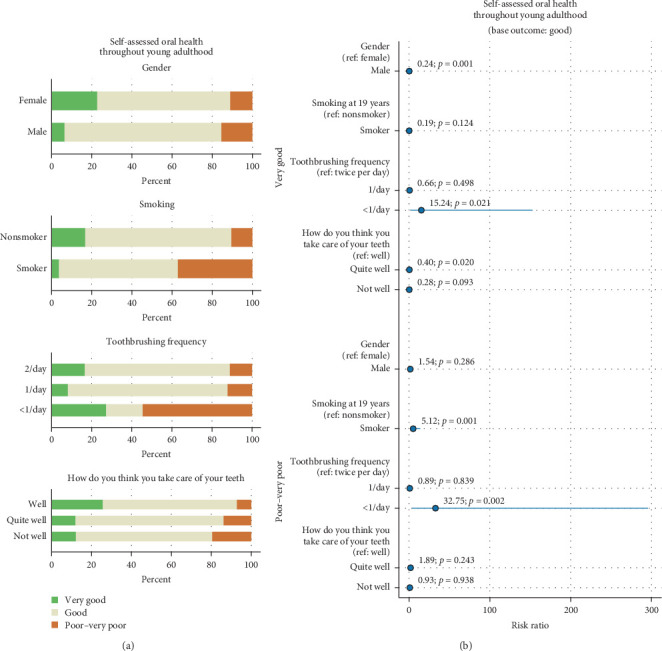
Distribution of self-assessed oral health by gender, smoking, toothbrushing frequency, and “How do you think you take care of your teeth” (a), and OR with 95% CI from the multinomial logistic regression model (outcome: self-assessed oral health) (b).

Gender, smoking, toothbrushing frequency, and the perceived quality of own dental care were predictors of SA oral health. Females and those who reported taking good care of their teeth (“well”) at age 19 were more likely to report their oral health as very good in young adulthood. In contrast, smokers were more likely to rate their oral health as poor or very poor. Among individuals who brushed their teeth less than once per day at age 19, SA oral health was polarized, resulting in a higher likelihood for both poor–very poor and very good SA oral health (Figure 3 and Table A6).

## 4. Discussion

The purpose of the present study was to identify predictors associated with oral health in young adulthood. Attitude towards oral health at age 19 years was associated with good PA oral health in young adults. Gender, smoking, toothbrushing frequency, and the perceived quality of own dental care were predictors of SA oral health.

In our study, SA oral health aligned with PA only to a certain degree. On one hand, as little as 30% of individuals with very good SA oral health presented with good PA oral health. Discrepancies between self-reported and clinically assessed oral status have been previously reported, particularly among individuals with disease [22]. On the other hand, 14% of those with poor to very poor SA oral health did meet the criteria for good PA oral health. It is plausible that young people base their judgement on factors not taken into account in the present study. These may include functional aspects, such as malocclusion and temporomandibular disorders, but also other factors which are not captured in a clinical examination, such as those related to psychological and social well-being [9, 23].

Our findings support the notion that a positive attitude towards oral health during younger years may contribute to maintaining good oral health over time. It is reasonable to assume that a positive oral health-related attitude would translate into positive oral health-related behaviors (e.g., good oral hygiene habits). In fact, it was found that the perceived importance of fluoride toothpaste and interproximal cleaning as well as the self-evaluated quality of own dental care were relevant for oral health in young adulthood. While oral health promotion programs aimed at adolescents have been shown to have positive behavioral effects [24], it has also been reported that oral health beliefs and behaviors tend to shift over late adolescence and young adulthood [25, 26]. When positive oral health-related beliefs persist, however, a beneficial long-term impact on caries activity and periodontal health has been shown [26]. Thus, reinforcing positive attitudes and behaviors may be particularly important to achieve and maintain good oral health throughout life. In this sense, it would be interesting to evaluate the impact of such a reinforcement on longitudinal trends in oral disease, such as changes in caries incidence in a long-term perspective. It should be noted that our population consisted mostly of regular attenders within the public dental service.

Other relevant predictors were identified in the present study. In the original cross-sectional dataset, smoking at age 19 years was associated with poorer oral health-related attitudes and behaviors [20]. A decade later, smoking was linked with poorer SA oral health, a higher risk for periodontitis and higher DFT scores. Similarly, the detrimental effect of smoking on periodontal health was already evident in young adults in the Dunedin study [27]. These findings align with current consensus as smoking is a recognized risk factor for periodontitis [8]. Regarding the association with caries, a systematic review by Jiang et al. [28] including 11 studies found smoking to be associated with an increased risk for caries.

The observation that males were at higher risk for periodontitis is also in agreement with previous reports [29]. Gender differences in self-rated oral health have been less consistently reported, with some authors reporting differences [30] and others finding none [23]. Our finding of an association between education levels at 19 years and DFT in young adulthood is in agreement with previous evidence. For instance, a systematic review by Schwendicke et al. [31], including 83 studies, found that lower level of parental or personal education were associated with a higher risk for caries in children/adolescents and adults. One seemingly contradictory finding was the fact that infrequent tooth brushing increased the likelihood for both poor–very poor but also very good SA oral health. Perhaps this is explained by a lack of disease awareness.

The use of register-based data allowed us to include a majority of the initially examined individuals despite the fact that 20% had moved to other areas of Sweden (data not shown). However, we were limited to those attending dental clinics reporting to SKaPa, which may have introduced a bias and, thereby limited the generalizability of our findings. The data originate from registrations by a large number of noncalibrated examiners, which could represent another source of bias. This may be particularly relevant for the self-reported oral health outcome, which is based on a question asked during routine clinical examination (by clinicians with varying degrees of training, experience, and motivation) rather than a controlled study environment. Furthermore, we were restricted to the data available in the registry, which implies a lack information on relevant confounders, such as diet or measures of oral hygiene, as well as on certain clinical parameters, such as bleeding on probing and clinical attachment levels.

## 5. Conclusion

Attitude towards one's own oral health at a young age was the dominant predictor of PA oral health in young adulthood. Gender, smoking, toothbrushing frequency, and the perceived quality of own dental care in late adolescence were significant predictors of SA oral health. Thus, establishing positive oral health-related attitudes and behavior in adolescence is beneficial for maintaining good oral health in a long-term perspective.

## Figures and Tables

**Table 1:** Characteristics at age 19 and 30–31 years^d^.

Variable	*N*	%
Gender		
Female	189	(56.4%)
Male	146	(43.6%)
At 19 years		
Parent education		
Upto lower secondary education	29	(8.7%)
Upper secondary education	135	(40.3%)
Post-secondary to tertiary education	119	(35.5%)
Not known	52	(15.5%)
Current education/occupation		
Theoretical upper secondary education	157	(46.9%)
Practical upper secondary education	155	(46.3%)
Working or other	23	(6.9%)
Tobacco smoking^b^	36	(10.8%)
** **Snuff use^b^	42	(12.5%)
Regular physical activity (at least once per week)^b^	224	(66.9%)
Toothbrushing (frequency)^b^		
Twice per day	265	(79.1%)
Once per day	58	(17.3%)
Several times per week	11	(3.3%)
Once a week or less	1	(0.3%)
Interdental cleaning (frequency)^b^		
Daily	19	(5.7%)
At least once per week	85	(25.4%)
Several times per month	103	(30.7%)
Rarely or never	128	(38.2%)
How do you think you take care of your teeth?^c^		
Well	80	(23.9%)
Quite well	206	(61.5%)
Not well	49	(14.6%)
How important is oral health to you?^a^		
Very important	250	(74.6%)
Quite important	81	(24.2%)
Not particularly important	4	(1.2%)
Not important at all	0	—
Importance of cleaning teeth^a^		
Very important	203	(60.6%)
Quite important	125	(37.3%)
Not particularly important	6	(1.8%)
Not important at all	1	(0.3%)
Importance of using fluoride toothpaste^c^		
Very important	148	(44.2%)
Quite important	128	(38.2%)
Not particularly important	47	(14.0%)
Not important at all	12	(3.6%)
Importance of interdental cleaning^c^		
Very important	29	(8.7%)
Quite important	105	(31.3%)
Not particularly important	157	(46.9%)
Not important at all	44	(13.1%)
Self-efficacy score (median [IQR])	30	[5]
How do you evaluate your possibility to impact your oral health?^c^		
Large	173	(51.6%)
Quite large	149	(44.5%)
Quite small	12	(3.6%)
Small	1	(0.3%)
Dental anxiety (categories according to dental anxiety score)		
Score ≤7	221	(66.0%)
Score 8–12	89	(26.6%)
Score ≥13	25	(7.5%)
At 30–31 years		
Residence (*N* = 334)		
East Sweden	37	(11.1%)
South Sweden	289	(86.5%)
North Sweden	8	(2.4%)
Employed	290	(86.6%)
Education (*N* = 334)		
Up to lower secondary education	10	(3.0%)
Upper secondary to post-secondary education <2 years	162	(48.5%)
Post-secondary ≥2 years to tertiary education	162	(48.5%)
Civil status (*N* = 334)		
Single	225	(67.4%)
Married	103	(30.8%)
Divorced	6	(1.8%)
Had children (*N* = 334)	197	(59.0%)
Disposable income in relation to living standard^e^ (in percentage; median [IQR]; *N* = 332)	262	[93]

*Note:* Patient-reported items considered to be reflective of ^a^attitudes, ^b^behaviors, and ^c^perceptions.

^d^ *N* = 335 unless specified otherwise.

^e^From STATIV, Statistics Sweden; family income after tax in relation to an official reasonable standard of living.

**Table 2:** Characteristics at age 19 and 30-31years by professionally-assessed oral health in young adulthood^a^.

Variables	Caries and/or periodontitis	Free from caries/periodontitis
Gender		
Female	162 (54.5%)	27 (71.1%)
Male	135 (45.5%)	11 (28.9%)
At 19 years		
Parents' education		
Upto lower secondary education	23 (7.7%)	6 (15.8%)
Upper secondary education	122 (41.1%)	13 (34.2%)
Post-secondary to tertiary education	104 (35.0%)	15 (39.5%)
Not known	48 (16.2%)	4 (10.5%)
Education at 19 years		
Theoretical upper secondary education	134 (45.1%)	23 (60.5%)
Practical upper secondary education	143 (48.1%)	12 (31.6%)
Working or other	20 (6.7%)	3 (7.9%)
Tobbaco smoking	34 (11.4%)	2 (5.3%)
Snuff use	40 (13.5%)	2 (5.3%)
Regular physical activity (at least once per week)	197 (66.3%)	27 (71.1%)
Toothbrushing (frequency)		
Twice per day	232 (78.1%)	33 (86.8%)
Once per day	54 (18.2%)	4 (10.5%)
Several times per week	10 (3.4%)	1 (2.6%)
Once a week or less	1 (0.3%)	0 (0.0%)
Interdental cleaning (frequency)		
Daily	16 (5.4%)	3 (7.9%)
At least once per week	78 (26.3%)	7 (18.4%)
Several times per month	92 (31.0%)	11 (28.9%)
Rarely or never	111 (37.4%)	17 (44.7%)
How do you think you take care of your teeth?		
Well	67 (22.6%)	13 (34.2%)
Quite well	183 (61.6%)	23 (60.5%)
Not well	47 (15.8%)	2 (5.3%)
How important is oral health to you?		
Very important	216 (72.7%)	34 (89.5%)
Quite important	77 (25.9%)	4 (10.5%)
Not particularly important	4 (1.3%)	0 (0.0%)
Importance of cleaning teeth		
Very important	175 (58.9%)	28 (73.7%)
Quite important	116 (39.1%)	9 (23.7%)
Not particularly important	6 (2.0%)	0 (0.0%)
Not important at all	0 (0.0%)	1 (2.6%)
Importance of using fluoride toothpaste		
Very important	131 (44.1%)	17 (44.7%)
Quite important	111 (37.4%)	17 (44.7%)
Not particularly important	43 (14.5%)	4 (10.5%)
Not important at all	12 (4.0%)	0 (0.0%)
Importance of interdental cleaning		
Very important	26 (8.8%)	3 (7.9%)
Quite important	91 (30.6%)	14 (36.8%)
Not particularly important	137 (46.1%)	20 (52.6%)
Not important at all	43 (14.5%)	1 (2.6%)
Self-efficacy score (median [IQR])	30 [6]	29 [4]
How do you evaluate your possibility to impact your oral health?		
Large	151 (50.8%)	22 (57.9%)
Quite large	135 (45.5%)	14 (36.8%)
Quite small	11 (3.7%)	1 (2.6%)
Small	0 (0.0%)	1 (2.6%)
Dental anxiety (categories according to dental anxiety score)		
Score ≤7	193 (65.0%)	28 (73.7%)
Score 8–12	81 (27.3%)	8 (21.1%)
Score ≥13	23 (7.7%)	2 (5.3%)
At 30–31 years		
Residence (*N* = 334)		
East Sweden	30 (10.1%)	7 (18.4%)
South Sweden	259 (87.5%)	30 (78.9%)
North Sweden	7 (2.4%)	1 (2.6%)
Employed	256 (86.2%)	34 (89.5%)
Education (*N* = 334)		
Upto lower secondary education	9 (3.0%)	1 (2.6%)
Upper secondary to post-secondary education <2 years	147 (49.7%)	15 (39.5%)
Post-secondary ≥2 years to tertiary education	140 (47.3%)	22 (57.9%)
Civil status (*N* = 334)		
Single	94 (31.8%)	9 (23.7%)
Married	199 (67.2%)	26 (68.4%)
Divorced	3 (1.0%)	3 (7.9%)
Had children (*N* = 334)	175 (59.1%)	22 (57.9%)
Disposable income in relation to living standard (median [IQR]; *N* = 332)	262 [92]	260 [111]

^a^ *N* = 335, unless specified otherwise.

**Table 3:** Characteristics at age 19 and 30–31 years by self-assessed oral health in young adulthood^a^.

Variables	Very good	Good	Poor–very poor
Gender			
Female	35 (81.4%)	102 (51.5%)	17 (47.2%)
Male	8 (18.6%)	96 (48.5%)	19 (52.8%)
At 19 years			
Parent education			
Upto lower secondary education	6 (14.0%)	15 (7.6%)	3 (8.3%)
Upper secondary education	16 (37.2%)	85 (42.9%)	17 (47.2%)
Post-secondary to tertiary education	19 (44.2%)	65 (32.8%)	9 (25.0%)
Not known	2 (4.7%)	33 (16.7%)	7 (19.4%)
Current education/occupation			
Theoretical upper secondary education	20 (46.5%)	92 (46.5%)	10 (27.8%)
Practical upper secondary education	20 (46.5%)	96 (48.5%)	22 (61.1%)
Working or other	3 (7.0%)	10 (5.1%)	4 (11.1%)
Tobacco smoking	1 (2.3%)	16 (8.1%)	10 (27.8%)
Snuff use	4 (9.3%)	24 (12.1%)	7 (19.4%)
Regular physical activity (at least once per week)	32 (74.4%)	127 (64.1%)	26 (72.2%)
Toothbrushing (frequency)			
Twice per day	36 (83.7%)	157 (79.3%)	24 (66.7%)
Once per day	4 (9.3%)	39 (19.7%)	6 (16.7%)
Several times per week	3 (7.0%)	2 (1.0%)	5 (13.9%)
Once a week or less	0 (0.0%)	0 (0.0%)	1 (2.8%)
Interdental cleaning (frequency)			
Daily	4 (9.3%)	11 (5.6%)	1 (2.8%)
At least once per week	11 (25.6%)	55 (27.8%)	8 (22.2%)
Several times per month	17 (39.5%)	55 (27.8%)	11 (30.6%)
Rarely or never	11 (25.6%)	77 (38.9%)	16 (44.4%)
How do you think you take care of your teeth?			
Well	18 (41.9%)	47 (23.7%)	5 (13.9%)
Quite well	20 (46.5%)	123 (62.1%)	23 (63.9%)
Not well	5 (11.6%)	28 (14.1%)	8 (22.2%)
How important is oral health to you?			
Very important	37 (86.0%)	150 (75.8%)	23 (63.9%)
Quite important	4 (9.3%)	47 (23.7%)	13 (36.1%)
Not particularly important	2 (4.7%)	1 (0.5%)	0 (0.0%)
Importance of cleaning teeth			
Very important	31 (72.1%)	126 (63.6%)	15 (41.7%)
Quite important	9 (20.9%)	69 (34.8%)	21 (58.3%)
Not particularly important	2 (4.7%)	3 (1.5%)	0 (0.0%)
Not important at all	1 (2.3%)	0 (0.0%)	0 (0.0%)
Importance of using fluoride toothpaste			
Very important	20 (46.5%)	89 (44.9%)	17 (47.2%)
Quite important	15 (34.9%)	72 (36.4%)	13 (36.1%)
Not particularly important	7 (16.3%)	30 (15.2%)	3 (8.3%)
Not important at all	1 (2.3%)	7 (3.5%)	3 (8.3%)
Importance of interdental cleaning			
Very important	4 (9.3%)	13 (6.6%)	5 (13.9%)
Quite important	15 (34.9%)	66 (33.3%)	11 (30.6%)
Not particularly important	21 (48.8%)	91 (46.0%)	13 (36.1%)
Not important at all	3 (7.0%)	28 (14.1%)	7 (19.4%)
Self-efficacy score (median [IQR])	30 [5]	29 [5]	30 [6]
How do you evaluate your possibility to impact your oral health?			
Large	25 (58.1%)	105 (53.0%)	21 (58.3%)
Quite large	17 (39.5%)	84 (42.4%)	14 (38.9%)
Quite small	1 (2.3%)	9 (4.5%)	1 (2.8%)
Small	—	—	—
Dental anxiety (categories according to dental anxiety score)			
Score ≤7	32 (74.4%)	125 (63.1%)	23 (63.9%)
Score 8–12	9 (20.9%)	58 (29.3%)	7 (19.4%)
Score ≥13	2 (4.7%)	15 (7.6%)	6 (16.7%)
At 30–31 years			
Residence (*N* = 276)			
East Sweden	2 (4.7%)	9 (4.6%)	4 (11.1%)
South Sweden	40 (93.0%)	182 (92.4%)	31 (86.1%)
North Sweden	1 (2.3%)	6 (3.0%)	1 (2.8%)
Employed	37 (86.0%)	171 (86.4%)	32 (88.9%)
Education (*N* = 276)			
Upto lower secondary education	0 (0.0%)	5 (2.5%)	1 (2.8%)
Upper secondary to post-secondary education <2 years	18 (41.9%)	103 (52.3%)	25 (69.4%)
Post-secondary ≥2 years to tertiary education	25 (58.1%)	89 (45.2%)	10 (27.8%)
Civil status (*N* = 276)			
Single	17 (39.5%)	54 (27.4%)	12 (33.3%)
Married	25 (58.1%)	141 (71.6%)	24 (66.7%)
Divorced	1 (2.3%)	2 (1.0%)	0 (0.0%)
Had children (*N* = 276)	25 (58.1%)	121 (61.4%)	19 (52.8%)
Disposable income in relation to living standard (median [IQR]; *N* = 275)	265 [80]	258 [96]	273 [77]

^a^ *N* = 277 unless specified otherwise.

**Table 4:** Predictors of oral health (results from logistic regression analyses; *N* = 335).

	Simple models		Final model^a^	
	OR (95% CI)	*p*-Value	OR (95% CI)	*p*-Value
Gender (ref: female)	0.48 (0.23, 1.01)	0.052	—	—
Education parents (ref: upper secondary education)				
Upto lower secondary education	2.71 (0.92. 8.00)	0.070	—	—
Post-secondary to tertiary education	1.38 (0.63, 3.04)	0.425	—	—
Not known	0.76 (0.24, 2.46)	0.648	—	—
Education at 19 years (ref: theoretical secondary education)				
Practical secondary education	0.51 (0.24, 1.07)	0.074	—	—
Working or other	0.82 (0.22, 3.02)	0.771	—	—
Dental anxiety (ref: score ≤7)				
Score 8–12	0.69 (0.30, 1.59)	0.388	—	—
Score ≥13	0.60 (0.13, 2.68)	0.500	—	—
Snuff use (ref: no)	0.36 (0.08, 1.55)	0.171	—	—
Tobacco smoking (ref: nonsmoker)	0.40 (0.09, 1.75)	0.225	—	—
Regular physical activity (ref: no)	1.20 (0.57, 2.52)	0.639	—	—
Toothbrushing frequency (ref: twice per day)				
Once per day	0.53 (0.18, 1.55)	0.246	—	—
Less than once per day	0.57 (0.07, 4.60)	0.595	—	—
Interdental cleaning frequency (ref: daily)				
At least once per week	0.52 (0.12, 2.24)	0.379	—	—
Some times per month	0.68 (0.17, 2.72)	0.584	—	—
Rarely or never	0.86 (0.22, 3.27)	0.821	—	—
Importance of brushing teeth with fluoride toothpaste (ref: fairly important)				
Very important	0.88 (0.43, 1.81)	0.723	—	—
Not important	0.46 (0.15, 1.45)	0.185	—	—
Importance of using dental floss/toothpick (ref: fairly important)				
Very important	0.66 (0.17, 2.52)	0.540	—	—
Not important	0.70 (0.34, 1.46)	0.341	—	—
Importance of cleaning teeth (ref: very important)	0.50 (0.23, 1.07)	0.075	—	—
How do you think you take care of your teeth (ref: well)				
Quite well	0.62 (0.30, 1.30)	0.209	—	—
Not well	0.21 (0.04, 0.96)	0.045	—	—
How important is oral health? (ref: very important)	0.31 (0.11, 0.90)	0.032	**0.31 (0.11, 0.90)**	**0.032**
Possibility to impact own oral health? (ref: large)				
Quite large	0.72 (0.35, 1.47)	0.367	—	—
Quite small–small	1.23 (0.25, 5.96)	0.795	—	—
Self-efficacy score	0.95 (0.88, 1.03)	0.226	—	—

*Note:* All models adjusted for years of observation. The values are presented in bold to highlight statistical significance.

^a^Hosmer–Lemeshow goodness-of-fit test: *p*=0.626.

**Table 5:** Predictors of DFT (results from linear regression analyses; *N* = 335).

	Simple models		Final model^a^	
	Coefficient (95% CI)	*p*-Value	Coefficient (95% CI)	*p*-Value
Gender (ref: female)	−0.33 (−1.13, 0.48)	0.426	—	—
Education parents (ref: upper secondary education)				
Upto lower secondary education	−0.56 (−2.05, 0.94)	0.464	—	—
Post-secondary to tertiary education	−1.14 (−2.06, −0.23)	0.015	—	—
Not known	−0.24 (−1.43, 0.95)	0.692	—	—
Education at 19 years (ref: theoretical secondary education)				
Practical secondary education	1.30 (0.48, 2.12)	0.002	**1.18 (0.36, 1.99)**	**0.005**
Working or other	2.13 (0.52, 3.74)	0.010	**1.62 (0.01, 3.23)**	**0.048**
Dental anxiety (ref: score ≤7)				
Score 8–12	0.52 (−0.39, 1.43)	0.263	—	—
Score ≥13	1.68 (0.15, 3.21)	0.032	—	—
Snuff use (ref: no)	1.01 (−0.20, 2.21)	0.101	—	—
Tobacco smoking (ref: nonsmoker)	2.15 (0.88, 3.43)	0.001	**1.72 (0.44, 3.00)**	**0.009**
Regular physical activity (ref: no)	−0.09 (−0.95, 0.76)	0.830	—	—
Toothbrushing frequency (ref: twice per day)				
Once per day	1.54 (0.50, 2.59)	0.004	—	—
Less than once per day	2.03 (−0.11, 4.16)	0.063	—	—
Interdental cleaning frequency (ref: daily)				
At least once per week	0.75 (−1.10, 2.60)	0.424	—	—
Several times per month	−0.09 (−1.90, 1.73)	0.926	—	—
Rarely or never	−0.65 (−2.43, 1.14)	0.476	—	—
Importance of brushing teeth with fluoride toothpaste (ref: fairly important)				
Very important	0.65 (−0.24, 1.53)	0.150	—	—
Not important	0.57 (−0.58, 1.72)	0.332	—	—
Importance of using dental floss/toothpick (ref: fairly important)				
Very important	−0.21 (−1.75, 1.33)	0.786	—	—
Not important	−0.90 (−1.78,- 0.01)	0.047	—	—
Importance of cleaning teeth (ref: very important)	0.93 (0.12, 1.74)	0.025	—	—
How do you think you take care of your teeth (ref: well)				
Quite well	0.98 (0.03, 1.94)	0.044	**1.07 (0.13, 2.00)**	**0.025**
Not well	1.72 (0.41, 3.04)	0.010	**1.69 (0.40, 2.98)**	**0.010**
How important is oral health? (ref: very important)	1.16 (0.25, 2.07)	0.013	—	—
Possibility to impact own oral health? (ref: large)				
Quite large	−0.32 (−1.14, 0.50)	0.446	—	—
Quite small–small	−0.60 (−2.70, 1.51)	0.578	—	—
Self-efficacy score	0.03 (−0.07, 0.13)	0.514	—	—

*Note:* All models adjusted for years of observation. The values are presented in bold to highlight statistical significance.

**^a^** Adjusted R-squared: 0.07.

**Table 6:** Predictors of periodontal health (results from multinomial logistic regression analyses; base outcome: PPD ≤3 mm; *N* = 335).

	Simple models				Final model^a^			
	PPD 4–5 mm or ≥1 teeth with PPD ≥6 mm		≥2 Teeth with PPD ≥6 mm		PPD 4–5 mm or ≥1 teeth with PPD ≥6 mm		≥2 Teeth with PPD ≥6 mm	
	RR (95% CI)	*p*-Value	RR (95% CI)	*p*-Value	RR (95% CI)	*p*-Value	RR (95% CI)	*p*-Value
Gender (ref: female)	1.19 (0.74, 1.91)	0.471	2.30 (1.08, 4.92)	0.032	1.04 (0.63, 1.71)	0.869	**2.59** **(1.15, 5.83)**	**0.022**
Parent education (ref: upper secondary education)								
Upto lower secondary education	0.30 (0.11, 0.86)	0.026	1.27 (0.36, 4.48)	0.706	**—**	**—**	—	—
Post-secondary to tertiary education	1.08 (0.63, 1.83)	0.780	1.30 (0.54, 3.17)	0.557	—	—	—	—
Not known	1.02 (0.51, 2.06)	0.953	1.46 (0.49, 4.36)	0.492	—	—	—	—
Education at 19 years (ref: theoretical secondary education)								
Practical secondary education	0.76 (0.47, 1.23)	0.264	0.92 (0.42, 2.02)	0.834	—	—	—	—
Working or other	1.03 (0.39, 2.75)	0.946	1.39 (0.35, 5.57)	0.641	—	—	—	—
Dental anxiety (ref: Score ≤7)								
Score 8–12	1.39 (0.82, 2.35)	0.216	0.66 (0.25, 1.71)	0.388	—	—	—	—
Score ≥13	1.03 (0.42, 2.54)	0.940	0.69 (0.15, 3.24)	0.640	—	—	—	—
Snuff use (ref: no)	0.99 (0.49, 2.01)	0.977	1.27 (0.44, 3.63)	0.656	—	—	—	—
Tobacco smoking (ref: nonsmoker)	1.34 (0.61, 2.95)	0.464	2.83 (1.05, 7.61)	0.039	1.43 (0.63, 3.27)	0.395	**3.67** **(1.31, 10.31)**	**0.014**
Regular physical activity (ref: no)	0.72 (0.44, 1.17)	0.188	0.85 (0.38, 1.88)	0.691	—	—	—	—
Toothbrushing frequency (ref: twice per day)								
Once per day	1.41 (0.76, 2.61)	0.280	1.96 (0.79, 4.85)	0.146	—	—	—	—
Less than once per day	1.27 (0.34, 4.79)	0.721	2.23 (0.42, 11.91)	0.348	—	—	—	—
Interdental cleaning frequency (ref: daily)								
At least once per week	1.21 (0.40, 3.66)	0.740	0.59 (0.10, 3.48)	0.559	—	—	—	—
Several times per month	1.03 (0.34, 3.09)	0.959	1.05 (0.20, 5.44)	0.954	—	—	—	—
Rarely or never	1.14 (0.39, 3.36)	0.811	1.21 (0.24, 6.04)	0.820	—	—	—	—

Importance of brushing teeth with fluoride toothpaste (ref: fairly important)								
Very important	1.89 (1.11, 3.21)	0.019	0.72 (0.31, 1.65)	0.435	**2.14** **(1.24, 3.70)**	**0.007**	0.72 (0.30, 1.70)	0.451
Not important	2.36 (1.19, 4.68)	0.014	1.10 (0.39, 3.10)	0.858	**2.25** **(1.12, 4.53)**	**0.023**	1.04 (0.36, 3.02)	0.946
Importance of using dental floss/toothpick (ref: fairly important)								
Very important	0.96 (0.36, 2.56)	0.927	1.08 (0.26, 4.37)	0.919	0.91 (0.33, 2.47)	0.854	1.11 (0.26, 4.72)	0.883
Not important	2.25 (1.32, 3.83)	0.003	1.42 (0.62, 3.27)	0.412	**2.39** **(1.37, 4.15)**	**0.002**	1.15 (0.48, 2.77)	0.753
Importance of cleaning teeth (ref: very important)	1.06 (0.65, 1.70)	0.824	1.34 (0.63, 2.83)	0.447	—	—	—	—
How do you think you take care of your teeth (ref: well)								
Quite well	0.94 (0.54, 1.63)	0.818	1.28 (0.48, 3.42)	0.617	—	—	—	—
Not well	1.24 (0.57, 2.69)	0.586	2.22 (0.67, 7.43)	0.194	—	—	—	—
How important is oral health? (ref: very important)	0.79 (0.46, 1.35)	0.387	0.85 (0.36, 2.01)	0.712	—	—	—	—
Possibility to impact own oral health? (ref: large)								
Quite large	1.00 (0.62, 1.60)	0.985	1.70 (0.78, 3.68)	0.181	—	—	—	—
Quite small–small	1.89 (0.54, 6.60)	0.316	2.98 (0.52, 16.96)	0.219	—	—	-	-
Self-efficacy score	1.02 (0.96, 1.08)	0.458	1.07 (0.98, 1.18)	0.135	—	—	—	—

*Note:* All models adjusted for years of observation. The values are presented in bold to highlight statistical significance.

^a^Hosmer–Lemeshow goodness-of-fit test: *p*=0.830.

**Table 7:** Predictors of self-assessed oral health (results from multinomial logistic regression analyses; base outcome: good; *N* = 277).

	Simple models				Final model^a^			
	Very good		Poor–very poor		Very good		Poor–very poor	
	RR (95% CI)	*p*-Value	RR (95% CI)	*p*-Value	RR (95% CI)	*p*-Value	RR (95% CI)	*p*-Value
Gender (ref: female)	0.25 (0.11, 0.56)	0.001	1.15 (0.56, 2.35)	0.705	**0.24** **(0.11, 0.57)**	**0.001**	1.54 (0.70, 3.40)	0.286
Parent education (ref: upper secondary education)								
Upto lower secondary education	2.06 (0 0.69, 6.14)	0.192	1.03 (0.27, 3.97)	0.964	—	—	—	—
Post-secondary to tertiary education	1.57 (0.75, 3.29)	0.234	0.69 (0.29, 1.64)	0.398	—	—	—	—
Not known	0.34 (0.07, 1.55)	0.163	1.00 (0.38, 2.67)	0.995	—	—	—	—
Education at 19 years (ref: theoretical secondary education)								
Practical secondary education	0.91 (0.46, 1.81)	0.796	2.23 (0.99, 5.01)	0.053	—	—	—	—
Working or other	1.60 (0.39, 6.51)	0.512	3.29 (0.85, 12.69)	0.084	—	—	—	—
Dental anxiety (ref: score ≤7)								
Score 8–12	0.58 (0.26, 1.31)	0.191	0.67 (0.27, 1.67)	0.394	-	—	—	—
Score ≥13	0.54 (0.12, 2.48)	0.425	2.11 (0.74, 6.03)	0.165	—	—	—	—
Snuff use (ref: no)	0.76 (0.25, 2.33)	0.634	1.72 (0.68, 4.36)	0.255	—	—	—	—
Tobaco smoking (ref: nonsmoker)	0.28 (0.04, 2.18)	0.225	4.24 (1.73, 10.39)	0.002	0.19 (0.02, 1.57)	0.124	**5.12** **(1.89, 13.85)**	**0.001**
Regular physical activity (ref: no)	1.69 (0.80, 3.58)	0.167	1.40 (0.63, 3.08)	0.405	—	—	—	—
Toothbrushing frequency (ref: twice per day)								
Once per day	0.44 (0.15, 1.31)	0.140	1.01 (0.39, 2.63)	0.988	0.66 (0.20, 2.17)	0.498	0.89 (0.31, 2.62)	0.839
Less than once per day	8.86 (1.34, 58.71)	0.024	19.10 (3.48, 104.84)	0.001	**15.24** **(1.52, 152.77)**	**0.021**	**32.75** **(3.63, 295.53)**	**0.002**
Interdental cleaning frequency (ref: daily)								
At least once per week	0.52 (0.14, 1.94)	0.327	1.68 (0.19, 14.87)	0.642	—	—	—	—
Several times per month	0.80 (0.22, 2.86)	0.727	2.31 (0.27, 19.87)	0.445	—	—	—	—
Rarely or never	0.38 (0.10, 1.42)	0.149	2.32 (0.28, 19.28)	0.437	—	—	—	—
Importance of brushing teeth with fluoride toothpaste (ref: fairly important)								
Very important	1.06 (0.50, 2.21)	0.887	1.09 (0.49 - 2.40)	0.831	—	—	—	—
Not important	1.08 (0.42, 2.80)	0.870	0.86 (0.30 - 2.47)	0.781	—	—	—	—
Importance of using dental floss/toothpick (ref: fairly important)								
Very important	1.44 (0.41, 5.09)	0.568	2.15 (0.63, 7.33)	0.220	—	—	—	—
Not important	0.94 (0.46, 1.93)	0.865	0.95 (0.42, 2.12)	0.892	—	—	—	—
Importance of cleaning teeth (ref: very important)	0.70 (0.34, 1.44)	0.328	2.40 (1.16, 4.95)	0.018	—	—	—	—
How do you think you take care of your teeth (ref: well)								
Quite well	0.42 (0.20, 0.87)	0.019	1.77 (0.64, 4.95)	0.274	**0.40** **(0.19, 0.87)**	**0.020**	1.89 (0.65, 5.49)	0.243
Not well	0.48 (0.16, 1.43)	0.186	2.63 (0.78, 8.86)	0.119	0.28 (0.06, 1.23)	0.093	0.93 (0.17, 5.14)	0.938
How important is oral health? (ref: very important)	0.52 (0.20, 1.30)	0.160	1.74 (0.82, 3.71)	0.150	—	—	—	—
Possibility to impact own oral health? (ref: large)								
Quite large	0.83 (0.42, 1.64)	0.594	0.86 (0.41 - 1.79)	0.681	—	—	—	—
Quite small– small	0.48 (0.06, 4.00)	0.499	0.54 (0.06, 4.51)	0.569	—	—	—	—
Self-efficacy score	1.02 (0.94, 1.11)	0.649	0.94 (0.87, 1.03)	0.178	—	—	—	—

*Note:* All models adjusted for years of observation. The values are presented in bold to highlight statistical significance.

^a^Hosmer–Lemeshow goodness-of-fit test: *p*=0.994.

## Data Availability

The data that support the findings of this study are available from the corresponding author upon reasonable request.
